# Transitions in the Swedish school system and the impact on student’s positive self-reported-health

**DOI:** 10.1186/1471-2458-14-1045

**Published:** 2014-10-07

**Authors:** Malin Rising Holmström, Niclas Olofsson, Kenneth Asplund, Lisbeth Kristiansen

**Affiliations:** Department of Nursing Sciences, Mid Sweden University, Holmgatan 10, 851 70 Sundsvall, Sweden; Research and development, County Council Västernorrland, Storgatan 1, 871 85 Härnösand, Sweden

**Keywords:** Health dialogue, Health promotion, Longitudinal, School nurse

## Abstract

**Background:**

To explore three school based transitions and their impact on positive self- reported- health (SRH), pre-school to elementary school (6–10 y), elementary school to junior high school (10-13y), and junior high school to upper secondary school/high school (13-16y), in a long-term longitudinal population based study.

**Methods:**

The study followed three cohorts through one school transition each.

A longitudinal study with data from 6693 Health Dialogue questionnaires were used. Data were collected in the middle of Sweden during 2007–2012 with school children age 6–16 years old.

**Results:**

Several significant factors were identified with an impact for a positive self-reported-health among children age 6-16y; not feeling sad or depressed, afraid or worried, positive school environment (schoolyard and restrooms), not bullied, good sleep, daily physical activity and ability to concentrate. There was no single factor identified, the factors differed according to gender and age.

**Conclusion:**

The study have identified several gender and age specific factors for successful school transitions relevant for a positive SRH. This is valuable information for school staff, parents and school children and provides a possibility to provide support and assistance when needed.

**Electronic supplementary material:**

The online version of this article (doi:10.1186/1471-2458-14-1045) contains supplementary material, which is available to authorized users.

## Background

School children experience several transitions during their progress through the educational system: first from home to preschool, then to elementary, middle and secondary schools, university/college and finally employment. Parallel to these school transitions children undergo natural development process (aging and growth) marked by considerable individual physical, intellectual and emotional change, as well as the socialization processes of learning and adapting to function as part of a group or school class [1, 2]. Meleis describes transition as a period between two relatively stable states, as a process of adaptation and habituation to a new situation or as “to be somewhere in between”, which implies a lack of control and belonging and that definition is used in this study. Furthermore way how individuals experience and perceive the transition process could be crucial to transition progression and outcome [3]. Transition within the school system could be viewed as a potential point at which a child’s future is decided. School system transitions usually progress from pre- and elementary schools, typically small units close to home, with only a few teachers and where parents are often well known to the school staff, to middle school/junior high schools, which are often larger, located at a distance from home and requiring a daily commute, with less contact between parents and school staff. These transitions are part of a broader range of transitions that children experience during schooling, such as switching between teachers, room changes between lessons, moving between various social groups, increasing academic demands, responsibility and independence. Together with the physical, mental and social developmental changes accompanying the child-adolescent-adult transitions, school transitions can raise existential questions regarding identity and liberation from the adult world (Table 1).

**Table 1:** **Development process versus school environment**

Age period	Development task	School environment
Preschool 6 years	Relatively slow growth better control over their body	Smaller schools units close to home
		Close contact between the home school
	Muscle strength and motor skill	
	Body shape more like an adult	Playful learning not focus on performance
	Able to dress and feed themselves, tying shoelaces, manage visiting the restroom	
		Continuity of staff a few caregivers, teachers working closest to the children enables attachment
	Healthy, fewer infections	
	Sleep eat and excretion rhythms more stable resemble adult	
	Concrete thinking	
	Language 10000–15000 vocabulary words Understands some jokes	
	Role plays	
	Positive to try new things	
	Writing their name knows their family name, address	
Middle childhood 10 years	School adjustment	Large schools units, regrouping new larger classes longer school days
	Attendance, conduct	
	Learning to read, do arithmetic	Decreasing contact between the home and school
	Making friends	
	Following rules of society for moral and social behavior	Several teachers working with the children
	Development of abstract thinking	Less attachment, increasing school demands and workload, developing own responsibility
		More focus on school achievements and grading
Adolescent 13–16 years	Cognitive ability developed further	Even larger school units located at a distance from home requiring daily travels with busses
	Puberty gender differences girls two years ahead of boys	
	Identity development maximal Forming an identity, Who am I? What will become of me?	Decreasing contact between parents and school staff. Teachers for each subject, responsibility for equipment change rooms for every lesson, different groups for each class. Mentoring
	Friends substitute for family	
	Real emancipation from the family ongoing with experimentation of limits	
	Sexuality	Academic achievement and adjustment to adult world.
	Learning skills needed for higher education or work	
		Increasing workload homework and higher academic demands, taking responsibility for themselves.
	Social activities athletics clubs etc.	
	Close friendship within and across gender	
		Growing into a adult person more independent from family and friends.

Adolescents often turn away from their traditional family support and prefer to test their own abilities. Meanwhile the adolescents enter a period of emotional intensity, and will not become cognitively mature until 20–25 y [4–6]. The combination of these factors creates high demands upon adolescents, creating the need for a systematic approach for support and intervention in schools. Adolescent might perceive such an approach as slightly more “neutral”, thus making to more likely to be accepted and to have impact.

Changing of roles and positions and the accompanying uncertainty can be considered the greatest transition encountered by children. This transition was evident from Kvalsund’s study of 6^th^ grade school children. It was found that as children reach the peak of one social/school stage they are swiftly presented with an apparently much steeper and more demanding challenge [7]. Indeed, transitions can be one of the most difficult periods in a child’s life and children that undergo poor transitions have increased rates of poor emotional health, truancy and behavior problems [8]. Academic performance has also been shown to decrease during transitions, especially during the transition from middle to secondary school (often described as the toughest) [1, 8]. Moreover, an unhappy child may become disengaged or disruptive, may lose motivation to learn and enter a spiral downwards [7, 9]. In extreme cases that can lead to children dropping out of the school system, which has been shown to be associated with low health [8].

Compared to other countries, a larger proportion of Swedish pupils leave primary school without authorization to enter high school. Of these, male students who immigrated to Sweden after school starts and students with parents with only compulsory school or upper secondary education are overrepresented [10]. Similar problems exist in several other countries [11], with males and pupils from underprivileged families again overrepresented, while drug use has also been identified as increasing the risk of dropout [12]. However, no single factor can account for school dropout, rather it is a slow context dependent process [13].

Perceived security, academic success and feeling at ease in school have been shown to promote positive health and school performance [14]. As such, good transition becomes vital for ensuring children are secure and settled, and therefore better able to engage in school work. When investigating the student’s perspective of transitions, Topping [15] identified a sense of belonging as a key promoter of learning and academic achievement [15]. Indeed, it is well known among teachers and school staff that learning and health are strongly interdependent [14, 16, 17]. To address some of the lack of adequate measures of child to adolescents health [18, 19], educational research efforts are aiming to reduce the risk of school dropouts during transitions [1]. To achieve this it is important to identify and support the positive health factors which can impact on children’s maintained positive self rated health during transitions. To our knowledge there are no reported school based children’s positive self reported health (SRH) studies focused on the impact of school transitions.

The school nurses and teachers have experienced based professional knowledge that the school transitions are critical periods in life and as it may have impact for future positive health and therefore we have chosen to study three school transitions in the Swedish school system; pre-school to fourth grade elementary school; elementary school to seventh grade middle/secondary school; seventh grade middle/secondary school to first year of upper secondary school/high school.

The aim was to explore three school based transitions, following three different school cohorts and describe factors impact on positive self-reported health (SRH) after transitions, pre-school to elementary school (6-10y), elementary school to middle school/secondary school (10-13y), and middle school/secondary school to upper secondary school/high school (13–16), in a longitudinal population based study.

## Methods

### Context

In Sweden it is compulsory for all children age 7 to 16 to attend school, and although preschool class (at the age of 6) is not compulsory, practical all children attend. Therefore the Swedish educational system contains of six major systematic transitions: (1) home to preschool; (2) to primary school; (3) to middle/junior high school; (4) to secondary school; (5) to upper secondary/high school; (6) to university or college and finally to work. Schools are coeducational and the school system is public financed. All public schools (including meals) are free of charge. The Swedish School Health Services (SHS) and school nurses are based within schools and shares the school environment with the school children on a daily basis. The role of the school nurse is to monitor children’s development, preserving and improving their mental and physical health, to promote healthy lifestyles among the children throughout their school years and to foster a positive relationship based on availability, absence of chare and non-compulsory [20, 21]. One approach used by the SHS and school nurses in the county of Västernorrland is the Health Dialogue (HD) concept, which consists of three parts: (a) a HD questionnaire; (b) a meeting between children, parents (in the case of 6-year olds) and the school nurse, in which the HD questionnaire is used as a basis for dialogue and; (c) registration of the HD questionnaire results in the child’s medical record and in an epidemiological database (provided that the parents have given their written consent) [22]. The HD concept is offered to all school children at four occasions (at 6, 10, 13 and 16 y), and although content repeats, the questions, content and question numbers are reformulated to be age appropriate. The HD approach differs from other child health research approaches in that the HD concept originates from clinical practice and is conducted solely by the school nurse. The HD concept represents a cross-sectional snapshot of a child’s SRH but also allows longitudinal studies of child development throughout their schooling [23].

### HD section a: the HD questionnaire

The HD questionnaire is structured to a positive salutogenetic health promoting approach, consisting of health-related questions, each phrased in a positive manner, and covering physical, mental and social dimensions of health (see Additional file 1 for the questionnaires). The physical dimension includes nutrition (eating breakfast every day, drinking soft drinks once a week or less), somatic problems (allergies, headache, stomach ache, back/neck/shoulder pain), self reported health and sleep habits. The mental dimension includes well-being (feeling low/sad, worried/afraid, irritable/bad tempered and having an adult to talk to), school environment (satisfaction in school, having the ability to concentrate and work in peace, stress over school work) and bullying (knowledge about a friend being bullied and being bullied personally). The third dimension is social health and includes leisure (time spent watching TV and using computers) and physical activity, both in school and during leisure time (active participation in PE classes, daily physical activity, part time work, smoking habits at home, use of tobacco, alcohol and drugs). As well as these three principal dimensions, an additional section including six sub-questions addresses the physical school environment (e.g., perceptions of classrooms, school yard and restrooms). The questionnaire was distributed by the school nurse and answered by the school children using paper-and pencil, in case of the 6-year-olds together with the parents. The HD questionnaire includes measurement of growth and BMI [23].

### HD section b: the meeting

The school nurse measures the school children’s height and weight, and calculates BMI [24]. Then the nurse sits down with each child for a dialogue using the HD questionnaire as a basis. The HD concept is inspired by “Motivational Interviewing” (MI), a method based on meetings (>20 min) and focused on increasing motivation for change. This approach is suitable for children whose concentration and endurance may be limited [25, 26].

### HD section c: the registration

After conducting the a- and b- parts of the HD concept, the school nurse performs a registration of the results in the digital medical journal and in a national database (if parental consent has been given). The school nurse then communicates written and/or verbal feedback to the school children and parents, including the results of the physical measurements.

### Sample

**Figure 1:**
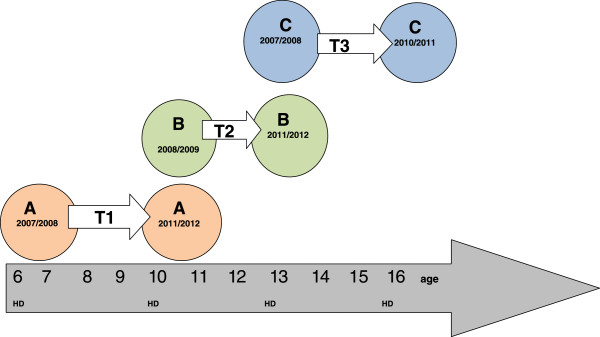
**Illustrating design of study and cohorts A,B,C.** It is the same individuals within the three cohorts but different individuals between the three cohorts. Cohort A pre-school (6y, 2007/2008) to fourth grade elementary school (10y, 2011/2012); cohort B fourth grade elementary school (10y, 2008/2009) to seventh grade middle/ secondary school (13 y, 2011/2012), and cohort C seventh grade middle/ secondary school (13y, 2007/2008) to first year of upper secondary school/high school (16y, 2010/2011). T= Transition T1 =preschool- elementary school, T2= elementary school- middle/ secondary school, T3= middle/ secondary school - upper secondary school/high school. HD= The Health Dialogue at 6,10,13 and 16 years of age. All HD’s has been conducted at two occasions with the same individuals and follows the academic year divided in two semesters and starts in the fall.

The data consists of all in all 6693 HD questionnaires conducted in the county of Västernorrland, Sweden during 2007–2012. All HD’s has been conducted at two occasions with the same individuals. Three cohorts has been created, cohort A pre-school (6y, 2007/2008) to fourth grade elementary school (10y, 2011/2012); cohort B fourth grade elementary school (10y, 2008/2009) to seventh grade middle/ secondary school (13 y, 2011/2012), and cohort C seventh grade middle/secondary school (13y, 2007/2008) to first year of upper secondary school/high school (16y, 2010/2011). It is the same individuals within the three cohorts but different individuals between the three cohorts (see Figure 1). All schools in the county were included and the county is characterized with large rural areas and a few cities; the county has approximately 250,000 inhabitants. For ethical reasons it is impossible to further investigate the dropouts or the reasons for dropouts.

Design of study and cohorts are illustrated in Figure 1 and distributions of the content in the cohorts and response rates are illustrated in Table 2.

**Table 2:** **Distribution and response rate in the three cohorts**

HD	Cohort A	Cohort B	Cohort C	Total
Year	2007-2012	2008-2012	2007-2011	
All	2187	2036	2470	6693
Response rate	1859 (85%)	1670 (82%)	1778 (72%)	5307 (79%)
Boys	948	852	889	2689
Girls	911	818	889	2618

### Ethics

Parental consent was granted for all HD’s in the study, which was approved by the Ethics Committee at the Medical Faculty, Umea University (no. 2008-122 M, 2013/91-31) and was conducted according to the ethical principles recommended by the Research Council.

### The variables

The approach of the HD was positive health promotive, and in accordance with this the variables have been coded and dichotomized consistently as positive (coded 1) and negative (coded 0). The dependent variable was determined to be self reported health, SRH, (My health is..) and was answered with the following five options: very good and rather good (coded 1); neither good/poor, rather poor, poor (coded 0). A model for each cohort A, B, C was created including the variables which showed significant differences in SRH between the two occasions, before and after the school transition (see Additional file 2 for variable coding). The cut off points of the variables were tested and validated (face validity) according to practice from several group discussions with school nurses in one of the municipals in the county.

### Statistical analyses

In order to assess the independent association between different positive variables in HD and SRH after school transitions in the three cohorts, the researchers controlled for the potential confounder gender and positive SRH before the transition. The first step of the analyses was to describe the prevalence of the different health factors among boys and girls in the three cohorts before and after the transitions (Table 3). Chi-2 test was conducted, and all positive health related variables that proved statistically significant were henceforward included in a model.

**Table 3:** **The percentage of positive SHR post transition in the three different school children cohorts in Västernorrland (Sweden), having not reported (NR) or having reported (R) the positive health characteristics measured before the pre transition in each cohort**

Positive health variables pre-transition	Girls NR	Cohort A ^1^	6-10 y Boys NR	Boys R	Girls NR	Cohort B ^2^	10-13 y Boys NR	Boys R	Girls NR	Cohort C ^3^	13-16 y Boys NR	Boys R
	N = 911	Girls R	N = 948		N = 918	Girls R	N = 952		N = 889	Girls R	N = 889	
Daily breakfast	94	96	97	96	93	91	92	96	76	91*	86	97
Positive school yard	90	91*	92	96	88	91	89	97*	61	91*	64	97
Positive rest rooms	72	96	71	96	63*	92*	49	97	49	90	46	98
Daily physical activity and play	81	96	86	97	64	88	60	98*	88	92*	87	97
Positve comfort	96	96	96	96*	96	90	92	96*	91	91*	90	97*
Positive concentration	39	99	33	98*	72	91	68	96	80	91	78	98*
Not stressed	N.A.	N.A.	N.A.	N.A.	N.A.	N.A.	N.A.	N.A.	57	91*	62	98*
Not bullied	70	97	58	98*	88	91	84	96	82	98*	85	97
No stomach ache	72	97*	78	97	70	91	75	97	66	90	83	97*
No back/neck/shoulder pain	94	96	95	96	80	90	78	96	68	92*	79	97*
Not sad or depressed	77	97*	79	98*	89*	92*	89	97*	73	92*	92	97*
Not worried or afraid	83	97*	85	97	90	91	92	96	87	92*	94	97*
Not irritated or bad mood	65	98*	56	98	75	91	71	96	54	92*	68	97
Positive sleep	96	97*	97	97	89	92*	86	96	84	91*	86	97*
No smoking home	95	97*	96	97	92	91	92	96	89	91*	88	97
Positive SRH	98	96	98	96	92	91	92	96*	88	93*	95	98*

1.2007/2008-2011/2012;2.2008/2009-2011/2012,3.2007/2008-2010/2011. * = p < = 0.05.

The multivariate analyses in the second step were conducted and the potential confounders were included in the analyses. A series of multiple-predictor models estimated the impact of earlier positive variable exposures on positive (SRH) outcomes in school transitions.

Multivariate analyses were conducted using logistic regression with a 95 percent confidence interval with dichotomized variables [27]. The statistical package SPSS version 21.0 was used for all statistical analyses.

## Results

The prevalence of school children reporting positive SRH in the three cohorts was relatively unchanged and remained stable at 88-98%, with small differences according to age and gender. Several of the positive health characteristics pre transition showed a statistical significant association to post transition SHR. Only the absence of sadness or feeling depressed were significantly associated pre transition to post transition SHR in all cohorts and within both girls and boys (Table 3). Eating breakfast daily or having tidy cleaned rest rooms pre transition were only associated with positive SHR post transition within the girls in cohort C and cohort B (Table 3).

Factors having a significant impact on positive SRH among children were identified in all cohorts. No single factor could account for changes in positive SRH during school transitions, but rather several factors depending on age and gender (Table 4).

**Table 4:** **Multivariate model of predictors of positive SRH over time (2007 to 2012) adjusted for gender (OR, Odds ratio with 95**% **confidence interval)**

All	Girls	Boys	All	Girls	Boys	All	Girls	Boys
6-10 y	6-10 y	6-10y	10-13 y	10-13 y	10-13y	13-16 y	13-16y	13-16 y
OR (CI)	OR (CI)	OR (CI)	OR (CI)	OR (CI)	OR (CI)	OR (CI)	OR (CI)	OR (CI)
No peers bullied* 1.9 (1.0-4.2)	No stomach ache 2.3 (0.8-6.6)	Not bullied 1.6 (0.6-4.3)	Not sad/depressed* 2.9 (1.7-5.2)	Not sad or depressed* 2.7 (1.4-5.5)	Not sad or depressed* 3.6 (1.1-12)	No headache 1.0 (0.7-1.6)	No back/neck/shoulder pain 0.8 (0.2-3.3)	No stomach ache 1.8 (0.6-5.6)
Not bullied 1.1 (0.5-2.5)	Not sad/depressed 0.9 (0.2-3.0)	Not sad/depressed* 2.0 (1.0-5.6)	Not worried/afraid 1.1 (0.6-2.3)	Positive sleep* 2.0 (1.0-4.2)	Positive comfort 2.0 (0.4-9)	No stomache ache 0.8 (0.5-1.3)	Not sad/ depressed 1.2 (0.4-3.3)	No back/neck/shoulder pain 0.5 (0.1-3.0)
No headache 1.0 (0.4-2.4)	Not worried/afraid 1.7 (0.5-5.2)	Positive concentration* 2.2 (1.0-6.1)	Positive sleep* 1.4 (1.0-2.6)	Positive rest rooms* 1.6 (1.0-2.8)	Daily PAP* 2.9 (1.0-10)	No back/neck/shoulder pain* 1.8 (1.2-2.7)	Not worried/afraid* 2.0 (1.0-6.4)	Not sad/depressed 0.8 (0.2-4.1)
No stomache ache 1.2 (0.5-2.7)	Not irritated/bad moody 1.8 (0.6-5.5)	Positive comfort* 3.8 (1.1-14)	Non smoking home* 1.8 (1.0-3.6)		Positive school yard 1.9 (0.5-7.1)	Not sad/depressed* 1.8 (1.0-3.0)	Not irritated/bad mood 1.4 (0.5-3.8)	Not worried/afraid 1.2 (0.3-4.4)
Not sad/depressed 1.1 (0.5-2.7)	Positive sleep* 4.2 (1.1-16)		Positive school yard* 1.9 (1.1-3.3)			Not worried/afraid 1.4 (0.7-2.5)	Not bullied 1.0 (0.3-2.6)	Not irritated/bad mood 0.9 (0.3-2.8)
Not worried/afraid* 1.9 (1.0-4.6)	Non smoking home* 4.2 (1.2-15)					Not irritated/bad mood 1.1 (0.7-1.7)	Positive comfort 1.2 (0.4-3.5)	Positive concentration* 2.8 (1.0-11)
Not irritated/bad mood 1.6 (0.7-3.5)	Positive school yard* 5.7 (2.0-17)					Not bullied 1.0 (0.6-1.6)	Sleep 0.5 (0.1-1.7)	Not stressed 2.4 (1.0-7.6)
Positive concentration 1.5 (0.7-3.4)						Positive concentration 1.2 (0.8-2.0)	Non smoking home 2.0 (0.1-28)	Positive comfort 0.6 (0.2-2.3)
Positive comfort* 2.2 (1.0-6.9)						Positive comfort* 1.5 (1.0-2.7)	Not stressed 1.0 (0.6-1.4)	Positive sleep* 2.6 (1.0-9.0)
Positive sleep* 3.5 (1.2-10)						Positive sleep 0.9 (0.5-1.5)	Daily PAP* 2.6 (1.0-7.2)	
Non smoking home* 2.4 (1.0-8.1)						Daily PAP* 1.6 (1.0-2.6)	Positive school yard* 2.3 (1.0-6.2)	
Positive school yard 1.1 (0.3-3.4)						Positive school yard* 1.1 (1.0-1.6)	Daily breakfast 1.3 (0.5-3.4)	
						Breakfast 1.1 (0.7-1.7)		

* = p < 0.0001.

### Cohort A

For all children age 6–10 y there appeared to be a link between experiencing positive sleep [3.5; 1.2-10], a non smoking home [2.4;1.0-8.1], feeling comfortable in school [2.2.0-6.9],not feeling afraid or worried [1.9;1.0-4-6], and not experienced friends bullied [1.9;1.0-4.2] and reporting a positive SRH in 4 th grade. An association could also be made between 6 y old girls experiencing the school yard positively [5.7; 2.0-17], a non smoking home [4.2;1.2-15], and positive sleep [4.2;1.1-16] and reporting a positive SRH as 10 y old, and among 6 y old boys feeling comfortable in school [3.8;1.1-14], 2) ability to concentration [2.2;1.0-6.1], 3) and not feeling sad or blue [2.0;1.0-5-6] and reporting a positive SRH (Table 4).

### Cohort B

These factors proved significant for all children age 10–13 y in order to report a positive SRH in 7 th grade, not feeling sad or blue [2.9;1.7-5.2], experience the school yard positive [1.9;1.1-3.3], a non smoking home [1.8; 1.0-3.6],and having positive sleep [1.4;1.0-2.6].

An association could also be seen between 10 y old girls not feeling sad or blue [2.7;1.4-5.5], having positive sleep [2.0;1.0-4.2], and positive experience of the schools rest rooms [1.6;1.0-2.8], for reporting a positive SRH as 13 y old, and among 10 y old boys not feeling sad or blue [3.6;1.1-12], and being physical active every day [2.9;1.0-10] for reporting a positive SRH (Table 4).

### Cohort C

These factors proved significant for all children age 13–16 y in order to report a positive SRH in the 1 st year of high school, not experiencing pains from back/neck/shoulders [1.8;1.2-2.7], not feeling sad or blue [1.8;1.0-3.0], daily physical active [1.6;1.0-2.6], feeling comfortable in school [1.5;1.0-2.7], positive experience of the school yard [1.1;1.0-1.6].

An association could also be seen made between 13 y old girls being daily physical active [2.6;1.0-7.2], experience the school yard positive [2.3;1.0-6.2], not feeling afraid or worried [2.0;1.0-6.4], for reporting a positive SRH as 16 y old, and among 13 y old boys experienced ability to cconcentrate [2.8;1.0-11], having a positive sleep [2.6;1.0-9.0], and reporting a positive SRH (Table 4).

## Discussion

The results show that the HD is able to identify factors impacting school children’s SRH during school transitions. Though analysis of self reported experiences of school life we have identified many significant factors relevant for SRH and learning, including being safe and secure at school, not feeling sad, not feeling afraid or worried, experiencing the schoolyard and restrooms positive (especially girls), not experiencing being bullied, having a good sleep, being physical active, having the ability to concentrate (especially boys).

As differences in the key factors were identified between the cohorts, this allows interpretation of how children’s age and development, as well as school organization and the pedagogical curriculum affect SRH.

### Feeling safe and secure

We interpreted that it was vital for children of 6–10 y to have the courage and inner security to experience a larger world, including a partial separating from parents. Furthermore, it is important that the schools physical and mental environment supports the children’s natural curiosity, without damaging the children’s trust.

Achieving a good start to school was found to be important for reporting positive SRH for 6 y old children.

### Experiencing social belonging

It seems as if this specific school transition for children 10–13 y bridges over a wider gap. We, therefore, claim that the social belonging is of great importance for these children. The school environment may provide opportunities to engage in interaction with other children, which again fosters feelings of social belonging and enhances pupil’s possibility of good school transition and to report positive SRH as 13 y old.

### Health

The health of 13-16y olds can be divided into several components such as healthy body, security within oneself and in relation to others. A positive SRH was significantly associated with a broad spectrum of variables reflecting physical, mental and social factors, and can be interpreted as good and robust self esteem being the key factor across positive physical, mental and social factors. Almost all variables had to have a positive outcome in order to report a positive SRH as this reflects a complex process in a sensitive period in children’s lives. This corresponds well with earlier research among adolescents which conceptualize health as a construct related to medical, psychological, social and lifestyle factors. Positive health ratings were affected in a similar manner to negative ratings, however, the absolute importance of hampering positive health may be greater because of the high prevalence of such ratings [28].

These findings identify health factors with a positive impact on SRH during transitions. These factors differed across ages and between genders, and thus contribute to the knowledge of the school nurses and support teams. This knowledge can now be applied to enhance facilitation of school children during school transitions, which will be vital in preventing school failure and dropouts. Other researchers have found that there is no single factor that can account for school dropout; rather it is a slow context dependent process [13]. Our findings suggest that it could allow school nurses, student health teams, health professionals, an opportunity to identify these factors and initiate intervention. The results could also contribute valuable information to the children themselves, as well as their families. Overall we found that children’s positive SRH is largely dependent on the school environment, which must be are experienced positively. This corresponds well with the results of Awartani et al. and Rising Holmström et al. [14, 29], which show that perceived security and to feel at ease within school are important for school success. Smoking remains one of the most important preventable causes of death in the world [30] and studies indicate that most adolescents begin using tobacco before 18 y old (as well as alcohol and other drugs). Indeed, rates of adolescent substance use are high in many Western countries, including Australia and the United States [31–33] and there are indications that some children begin tobacco use before 10 y of age [34].

The relationship between parental and peers smoking, family structure and parent–child relationship, and adolescent smoking indicate that all of these factors contribute to the onset of daily smoking in adolescents [35–38]. Shenghan Lai et al. [39] studied the association between cigarette smoking and drug abuse in the United States and suggest that cigarette smoking may act as a gateway drug to illegal drug use [39]. Developmental research shows that adolescent substance misuse (smoking, alcohol and drugs) can result in immediate and long-term health and behavioral problems [40], particularly substance dependence [41], mental and physical health problems [42] and disruption to family and social relationships [43]. To sleep well is important for school children and the association between inadequate sleep, wellbeing and various health problems have been well studied [44–47]. Our result shows that physical activity has an impact on positive SRH. This is in line with earlier research which established that physical activity generates positive effects on children’s health, including regular nutrition habits, normal weight and health development [48–50]. Considering that health and learning are strongly interdependent, this result has relevance for children’s learning ability and suggests that successful transitions require teamwork between children, parents, teachers, school staff and school nurses.

### Methodological considerations

This study was limited by the fact that all results are limited to three cohorts of Swedish school children; however, as a population based survey including all school children SRH from 6–16 years and the high total response rate (79%) provides considerable strength and increases the generalizability of the results. In all research concerning children it is always difficult to find methods that are valid and reliable and there has been criticism of the HD concept based on the assumption that young children’s self-assessment of their own health is invalid due to the maturity and level of development, and that small children cannot verbalize their own health, and are instead influenced by their parents and surroundings. Riley’s [51] study demonstrates adequate understanding, reliability and validity of child self reports at ages as low as 6, which increases after the age of 7 in general populations. Moreover, the reliability of reports by children (8–11 y old) was robust on health questionnaires developed especially for this age group [51]. In the HD concept questionnaires have been developed especially for each age group and includes images and visual estimation scales to enable and ensure the child’s participation. Furthermore, concrete examples and “thinking aloud” were used as strategies to interpret abstract concepts during meetings between the pupil and school nurse. Such an approach has been shown to be successful in research among children of 5 to 9 years old [52]. Earlier research has studied this and found that young children (6-year olds), have a concrete understanding of health that is focused on hurts, aches and nutrition [53, 54] and that children can reliably self-report their health [55]. While much effort has been invested into the development and validation of the HD questionnaire [56]. Furthermore the HD concept was developed and tested by end users (school nurses) and this effected the implementation as well as the response rate [57–59]. The HD was conducted in a systematically similar approach by school nurses within the county [22] and as it was repeated four times, enabled the school child and school nurse to establish a continuous health promotive relationship [22], contribution to efficient transition of gaps in the school system.

## Conclusion

Knowledge that different health factors during school children’s school transitions have impact for future positive self-reported-health, and that these health factors may vary depending on age and gender, adds vital knowledge to the different actors on the school arena.

## Electronic supplementary material

Additional file 1: **Health questionnaire for 6-,10-,13- and 16- year- olds (4 versions) in pdf.** (PDF 852 KB)

Additional file 2: **The coding of the variables in pdf.** (PDF 222 KB)

## Abbreviations

SRH: Self-reported-health
HD: The Health Dialogue concept
BMI: Body mass index
PE: Physical education
PAP: Physical activity and play.
