# Supplementary material for: Transitions in the Swedish school system and the impact on student’s positive self-reported-health
Source: BMC Public Health. 2014 Oct 7;14:1045. doi: 10.1186/1471-2458-14-1045 (PMC4210600; doi:10.1186/1471-2458-14-1045)
Supplement: Supplementary file 1 — Additional file 1: Health questionnaire for 6-,10-,13- and 16- year- olds (4 versions) in pdf. (PDF 852 KB) [file 12889_2013_7173_MOESM1_ESM.pdf]

Additional file 1
Health questionnaire for 6-,10-,13- and 16- year- olds.
The English version is still not semantically valid, but the work is ongoing.

Landstinget Västernorrland

HEALTH QUESTIONS IN PREPARATORY CLASS (6-Y)

Name:	Swedish personal ID no:

The school nurse fills this out:

Date of the health conversation:			
Height: cm	Weight: kg	BMI:	Sex:

Please answer the health questions with your parents.

The answers will be the basis for the health conversation with the school nurse.

WORKING ENVIRONMENT

1. I think that…	Very good	good	whether good or bad	bad	very bad
	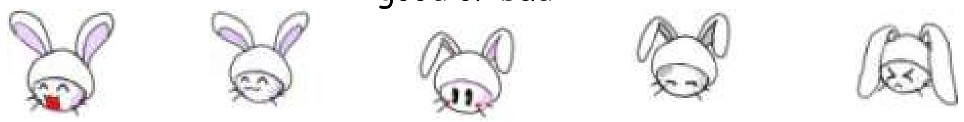				
a. the classrooms are	☐	☐	☐	☐	☐
b. the toilets are	☐	☐	☐	☐	☐
c. the playground is	☐	☐	☐	☐	☐
d. the sports hall	☐	☐	☐	☐	☐

is

e. the showers are	☐	☐	☐	☐	☐
f. the canteen is	☐	☐	☐	☐	☐

2. I can work undisturbed in school

☐ always ☐ often ☐ sometimes ☐ seldom ☐ never

3. I can concentrate in school

☐ always ☐ often ☐ sometimes ☐ seldom ☐ never

4. I know of someone at my school who has been bullied, excluded or treated badly in another way during the last three months (called names, pushed etc.)

	**no**	**yes**
a. by other pupils at school?	☐	☐
b. by adults at school?	☐	☐

5. I have been bullied, excluded or treated badly in another way during the last three months (called names, pushed, etc.)

	**no**	**yes**
a. by other pupils at school?	☐	☐
b. by adults at school?	☐	☐

6. I like it in school

**A lot**	**It's ok**	**It's whether good or bad**	**Not really**	**Not at all**
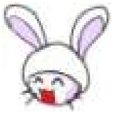	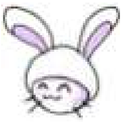	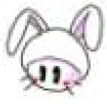	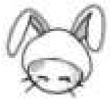	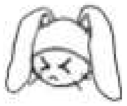
☐	☐	☐	☐	☐

EATING HABITS AND PHYSICAL ACTIVITIES

7. I have....

	every school day	3-4 school days a week	1-2 school days a week	never
a. breakfast	☐	☐	☐	☐
b. lunch	☐	☐	☐	☐
c. dinner	☐	☐	☐	☐

8. I drink sweetened drinks (soft drinks, juice, fruit drinks)

☐ never/seldom
☐ less than once a week
☐ 1-2 days a week
☐ 3-4 days a week
☐ 5 -7 days a week

9. I actively take part in gym class at school and climb, run, jump or play ball games

☐ always
☐ every other time
☐ a few times a month
☐ seldom
☐ never

SPARE TIME

10. In my spare time I play and move so that I get out of breath and warm

☐ 5 -7 times a week
☐ 3-4 times a week
☐ 1-2 time a week
☐ seldom
☐ never

11. In my spare time I sit in front of the TV/computer/with my mobile

☐ 2 hours or less per day
☐ 3 - 4 hours per day
☐ 5 - 6 hours per day
☐ more than 6 hours per day

12. Does anyone smoke inside the house where you live?

☐ seldom/ never
☐ **sometimes**
☐ **often**

PHYSICAL AND MENTAL HEALTH

13. I feel..

☐ very well	☐ well	☐ whether well or bad	☐ bad	☐ very bad

14. During the last three months

I have had a disturbing…	never	seldom	Some times	often	always
a. headache	☐	☐	☐	☐	☐
b. stomach ache	☐	☐	☐	☐	☐
c. pain in the back/ neck/shoulders	☐	☐	☐	☐	☐

15. During the last three months

I have felt…	never	seldom	Some times	often	always
a. sad or low	☐	☐	☐	☐	☐
b. worried or afraid	☐	☐	☐	☐	☐
c. irritated or in a bad mood	☐	☐	☐	☐	☐

16. I sleep well

☐ always	☐ often	☐ sometimes	☐ seldom	☐ never

HAVE YOU AND YOUR PARENTS GOT ANY QUESTIONS ABOUT THE BODY OR ANYTHING TO TELL ME ABOUT YOUR HEALTH?

____

____

____

Landstinget Västernorrland

HEALTH QUESTIONS IN THE 4TH GRADE

Name:	Swedish personal ID no:

The school nurse fills this out:

Date of the health conversation:			
Height: cm	Weight: kg	BMI:	Sex:

Tick the box that fits your opinion

WORKING ENVIRONMENT

1. I think that..	Very good	good	whether good or bad	bad	very bad
a. the classrooms are	☐	☐	☐	☐	☐
b. the toilets are	☐	☐	☐	☐	☐
c. the playground is	☐	☐	☐	☐	☐
d. the sports hall is	☐	☐	☐	☐	☐
e. the showers are	☐	☐	☐	☐	☐
f. the canteen is	☐	☐	☐	☐	☐

2. I can work undisturbed in school

☐ always ☐ often ☐ sometimes ☐ seldom ☐ never

3. I can concentrate during the lessons

☐ **always** ☐ **often** ☐ **sometimes** ☐ **seldom** ☐ **never**

4. I feel stressed over school work (means under pressure, a feeling of I won't make it in time)

☐ always ☐ seldom ☐ sometimes ☐ often ☐ always

5. I know of someone at my school who has been bullied, excluded or treated badly in another the last three months

**a. by other pupils at school?**	☐ no	☐ **yes, via Internet or mobile**	☐ **yes, in another way**	☐ **yes, via Internet or mobile and In another way**
**b. by adults at school?**	☐ no	☐ **yes, via Internet or mobile**	☐ **yes, in another way**	☐ **yes, via Internet or mobile and In another way**

c. If you answered yes on a or b or both a and b, please describe in what way……………………………

………

………………………………

6. I have been bullied, excluded or treated badly in another way during the last three months

a. **by other pupils at school?**	☐ no	☐ **yes, via Internet or mobile**	☐ **yes, in another way**	☐ **yes, via Internet or mobile and In another way**
b. **by adults at school?**	☐ no	☐ **yes, via Internet or mobile**	☐ **yes, in another way**	☐ **yes, via Internet or mobile and In another way**

c. If you answered yes on a or b or both a and b, please describe in what way ……………………………

………

7. I like it in school	**A lot**	**It's ok**	**It's whether good or bad**	**Not really**	Not at all
	☐	☐	☐	☐	☐

EATING HABITS AND PHYSICAL ACTIVITY

8. I have…	every school day	3-4 school days a week	1-2 school days a week	never
a. breakfast	☐	☐	☐	☐
b. lunch	☐	☐	☐	☐
c. dinner	☐	☐	☐	☐

9. I drink sweetened drinks (soft drinks, juice, fruit drinks)

☐ never/seldom ☐ less than once a week ☐ 1-2 days a week ☐ 3-4 days a week ☐ 5 – 7 days a week

10. I actively take part in gym class at school

☐ always ☐ every other time ☐ a few times a month ☐ seldom ☐ never

SPARE TIME

11. In my spare time I do sports/exercise/move in another way

☐ 5 -7 times a week ☐ 3-4 times a week ☐ 1-2 times a week ☐ seldom ☐ never

12. In my spare time I sit in front of the TV/computer/with my mobile

☐ 2 hours or less per day ☐ 3 - 4 hours per day ☐ 5 - 6 hours per day ☐ more than 6 hours per day

13. Does anyone smoke inside the house where you live?

☐ never ☐ seldom ☐ sometimes ☐ often

PHYSICAL AND MENTAL HEALTH

14. I feel…

☐ very well ☐ well ☐ whether well or bad ☐ bad ☐ very bad

15. During the last <u>three months</u> I have had a disturbing…	never	seldom	Some times	often	always
a. headache	☐	☐	☐	☐	☐
b. stomach ache	☐	☐	☐	☐	☐
c. pain in the back/ neck/shoulders	☐	☐	☐	☐	☐

16. During the last <u>three months</u> I have felt…	never	seldom	sometime	often	always
a. sad or low	☐	☐	☐	☐	☐
b. worried or afraid	☐	☐	☐	☐	☐
c. irritated or in a bad mood	☐	☐	☐	☐	☐

17. Have you got an adult with whom you can talk about what is important to you?

☐ yes ☐ no

18. I sleep well

☐ always ☐ often ☐ sometimes ☐ seldom ☐ never

QUESTIONS ABOUT THE BODY

At the health dialogue, you have the opportunity to talk about your body, like about how you have grown. Please feel free to write down if you have any questions about the way you feel or if you want to tell me something about your health:

___

THE FUTURE

This is the way I imagine my future as an adult. Please write it down here:

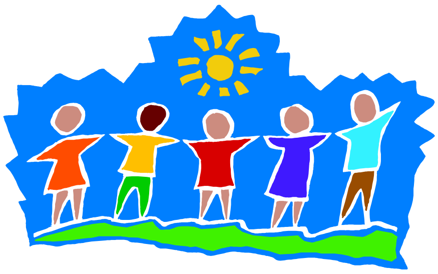

Landstinget Västernorrland

HEALTH QUESTIONS IN THE 7TH GRADE

Name:	Swedish personal ID no:

The school nurse fills this out:

Date of the health conversation:			
Height: cm	Weight: kg	BMI:	Sex:

Tick the box that fits your opinion

WORKING ENVIRONMENT

1. I think that..	Very good	good	whether good or bad	bad	very bad
a. the classrooms are	☐	☐	☐	☐	☐
b. the toilets are	☐	☐	☐	☐	☐
c. the playground is	☐	☐	☐	☐	☐
d. the sports hall is	☐	☐	☐	☐	☐
e. the showers are	☐	☐	☐	☐	☐
f. the canteen is	☐	☐	☐	☐	☐

2. I can work undisturbed in school

☐ always ☐ often ☐ sometimes ☐ seldom ☐ never

3. I can concentrate during the lessons

☐ always ☐ often ☐ sometimes ☐ seldom ☐ never

4. I feel stressed over school work (means under pressure, a feeling of I won't make it in time)

☐ never ☐ seldom ☐ sometimes ☐ often ☐ always

5. I know of someone at my school who has been bullied, excluded or treated badly in another the last three months

**a. by other pupils at school?**	☐ no	☐ **yes, via Internet or mobile**	☐ **yes, in another way**	☐ **yes, via Internet or mobile and In another way**
**b. by adults at school?**	☐ no	☐ **yes, via Internet or mobile**	☐ **yes, in another way**	☐ **yes, via Internet or mobile and In another way**

c. If you answered yes on a or b or both a and b, please describe in what way……………………………

……

………………………………

6. I have been bullied, excluded or treated badly in another way during the last three months

a. **by other pupils at school?**	☐ no	☐ **yes, via Internet or mobile**	☐ **yes, in another way**	☐ **yes, via Internet or mobile and In another way**
b. **by adults at school?**	☐ no	☐ **yes, via Internet or mobile**	☐ **yes, in another way**	☐ **yes, via Internet or mobile and In another way**

c. If you answered yes on a or b or both a and b, please describe in what way …………………………

……

7. I like it in school	**A lot**	**It's ok**	**It's whether good or bad**	**Not really**	Not at all
	☐	☐	☐	☐	☐

EATING HABITS AND PHYSICAL ACTIVITY

8. I have…

	every school day	3-4 school days a week	1-2 school days a week	never
a. breakfast	☐	☐	☐	☐
b. lunch	☐	☐	☐	☐
c. dinner	☐	☐	☐	☐

9. I drink sweetened drinks (soft drinks, juice, fruit drinks)

☐ never/seldom ☐ less than once a week ☐ 1-2 days a week ☐ 3-4 days a week ☐ 5 – 7 days a week

10. I actively take part in gym class at school

☐ always ☐ every other time ☐ a few times a month ☐ seldom ☐ never

SPARE TIME

11. In my spare time I do sports/exercise/move in another way

☐ 5 -7 times a week ☐ 3-4 times a week ☐ 1-2 times a week ☐ seldom ☐ never

12. In my spare time I sit in front of the TV/computer/with my mobile

☐ 2 hours or less per day ☐ 3 - 4 hours per day ☐ 5 - 6 hours per day ☐ more than 6 hours per day

13. Does anyone smoke inside the house where you live?

☐ never ☐ **seldom** ☐ **sometimes** ☐ **often**

PHYSICAL AND MENTAL HEALTH

14. I feel…

☐ very well ☐ well ☐ whether well ☐ bad ☐ very bad

or bad

15. During the last <u>three months</u> I have had a disturbing…	never	seldom	sometime s	often	always
a. headache	☐	☐	☐	☐	☐
b. stomach ache	☐	☐	☐	☐	☐
c. pain in the back/ neck/shoulders	☐	☐	☐	☐	☐

16. I use painkillers

☐ never ☐ a few times per year ☐ a few times per month ☐ a few times per week ☐ on a daily basis

17. During the last <u>three months</u> I have felt…	never	seldom	Some times	often	always
a. sad or low	☐	☐	☐	☐	☐
b. worried or afraid	☐	☐	☐	☐	☐
c. irritated or in a bad mood	☐	☐	☐	☐	☐

18. Have you got an adult with whom you can talk about what is important to you?

☐ yes ☐ no

19. I sleep well

☐ always ☐ often ☐ sometimes ☐ seldom ☐ never

ALCOHOL/DRUGS/TOBACCO

20. I …	never	have tested	once or a few times a month	once or a few times a week	on a daily basis
a. smoke	☐	☐	☐	☐	☐
b. take snuff	☐	☐	☐	☐	☐

21. I drink alcohol (medium-strong beer, strong beer, strong cider, wine, alcopops or spirit)

☐ **never** ☐ **have tested** ☐ **once/ a few times per year** ☐ **once/ a few times per month** ☐ **once/ a few times per week**

22. If I am offered drugs I say

☐ definitely no ☐ **probably no** ☐ **possible yes** ☐ **yes**

BODY DEVELOPMENT AND RELATIONSHIPS

Have you got any questions or anything to tell me about your body development, contraceptives or any other questions about feelings, sex or relationships?

☐ yes ☐ **no**

If yes, please write it down here:

____

____

____

THE FUTURE

This is the way I imagine my future as an adult. Please write it down here:

____

Landstinget Västernorrland

HEALTH QUESTIONS IN UPPER SECONDARY SCHOOL

Name:	Swedish personal ID no:

The school nurse fills this in:

Date of the health conversation:			
Height: cm	Weight: kg	BMI:	Sex:

Tick the box that fits your opinion

WORKING ENVIRONMENT

1. I think that	Very good	good	whether good or bad	bad	very bad
a. the classrooms are	☐	☐	☐	☐	☐
b. the toilets are	☐	☐	☐	☐	☐
c. the playground is	☐	☐	☐	☐	☐
d. the sports hall is	☐	☐	☐	☐	☐
e. the showers are	☐	☐	☐	☐	☐
f. the canteen is	☐	☐	☐	☐	☐

2. I can work undisturbed in school

☐ always ☐ often ☐ sometimes ☐ seldom ☐ never

3. I can concentrate during the lessons

☐ always ☐ often ☐ sometimes ☐ seldom ☐ never

4. I feel stressed over school work (means under pressure, a feeling of I won't make it in time)

☐ never ☐ seldom ☐ sometimes ☐ often ☐ always

5. I know of someone at my school who has been bullied, excluded or treated badly in another the last three months

**a. by other pupils at school?**	☐ no	☐ **yes, via Internet or mobile**	☐ **yes, in another way**	☐ **yes, via Internet or mobile and In another way**
**b. by adults at school?**	☐ no	☐ **yes, via Internet or mobile**	☐ **yes, in another way**	☐ **yes, via Internet or mobile and In another way**

c. If you answered yes on a or b or both a and b, please describe in what way………………………………

………

…………………………………

6. I have been bullied, excluded or treated badly in another way during the last three months

a. **by other pupils at school?**	☐ no	☐ **yes, via Internet or mobile**	☐ **yes, in another way**	☐ **yes, via Internet or mobile and In another way**
b. **by adults at school?**	☐ no	☐ **yes, via Internet or mobile**	☐ **yes, in another way**	☐ **yes, via Internet or mobile and In another way**

c. If you answered yes on a or b or both a and b, please describe in what way ……………………………

………

7. I like it in school	**A lot**	**It's ok**	**It's whether good or bad**	**Not really**	Not at all
	☐	☐	☐	☐	☐

EATING HABITS AND PHYSICAL ACTIVITY

8. I have…	every school day	3-4 school days a week	1-2 school days a week	never
a. breakfast	☐	☐	☐	☐
b. lunch	☐	☐	☐	☐

c. dinner ☐ ☐ ☐ ☐

9. I drink sweetened drinks (soft drinks, juice, fruit drinks)

☐ never/seldom ☐ less than once a week ☐ 1-2 days a week ☐ 3-4 days a week ☐ 5 – 7 days a week

10. I actively take part in gym class at school

☐ always ☐ every other time ☐ a few times a month ☐ seldom ☐ never

SPARE TIME

11. In my spare time I do sports/exercise/move in another way

☐ 5 -7 times a week ☐ 3-4 times a week ☐ 1-2 times a week ☐ seldom ☐ never

12. In my spare time I sit in front of the TV/computer/with my mobile

☐ 2 hours or less per day ☐ 3 - 4 hours per day ☐ 5 - 6 hours per day ☐ more than 6 hours per day

13. Do you have a job in your spare time?

☐ yes ☐ no

PHYSICAL AND MENTAL HEALTH

14. I feel…

☐ very well	☐ well	☐ whether well or bad	☐ bad	☐ very bad

15. During the last <u>three months</u> I have had a disturbing…	never	seldom	Some times	often	always
a. headache	☐	☐	☐	☐	☐
b. stomach ache	☐	☐	☐	☐	☐
c. pain in the back/ neck/shoulders	☐	☐	☐	☐	☐

16. I use painkillers

☐ never	☐ a few times per year	☐ a few times per month	☐ a few times per week	☐ on a daily basis

16. During the last <u>three months</u> I have felt…	never	seldom	some times	often	always
a. sad or low	☐	☐	☐	☐	☐
b. worried or afraid	☐	☐	☐	☐	☐
c. irritated or in a bad mood	☐	☐	☐	☐	☐

18. Have you got an adult with whom you can talk about what is important to you?

☐ yes	☐ no

19. I sleep well

☐ always	☐ often	☐ sometimes	☐ seldom	☐ never

ALCOHOL/DRUGS/TOBACCO

20. I ...	never	have tested	once or a few times a month	once or a few times a week	on a daily basis
a. smoke	☐	☐	☐	☐	☐
b. take snuff	☐	☐	☐	☐	☐

21. I drink alcohol (medium-strong beer, strong beer, strong cider, wine, alcopops or spirit)

☐ never ☐ have tested ☐ once/ a few times per year ☐ once/ a few times per month ☐ once/ a few times per week

22. If I am offered drugs I say

☐ definitely no ☐ probably no ☐ possible yes ☐ yes

BODY DEVELOPMENT AND RELATIONSHIPS

Have you got any questions or anything to tell me about your body development, contraceptives or any other questions about feelings, sex or relationships?

☐ yes ☐ no

If yes, please write it down here:

____

____

THE FUTURE

This is the way I imagine my future as an adult. Please write it down here:

____

____
