# Supplementary material for: Transitions in the Swedish school system and the impact on student’s positive self-reported-health
Source: BMC Public Health. 2014 Oct 7;14:1045. doi: 10.1186/1471-2458-14-1045 (PMC4210600; doi:10.1186/1471-2458-14-1045)
Supplement: Supplementary file 2 — Additional file 2: The coding of the variables in pdf. (PDF 222 KB) [file 12889_2013_7173_MOESM2_ESM.pdf]

Additional file 2

A model for each cohort A, B, C was created consisting of variables according to Table 3 and 4, coded as follows. The cut off points of the variables were tested and validated according to practice in a group discussion with school nurses in the county.

Perceptions about the school environment was assessed by asking how the school children find the school yard (**1**) and restrooms (**2**) (I find the…), answered in five options including very good, good, neither good nor poor (coded 1), poor and very poor (coded 0; **3**). The ability to concentration was assessed by asking for occurrence of concentration (I can concentrate in school) answered in five options including always, often (coded 1), sometimes, seldom, and never (coded 0); **4**) occurrence of stress about schoolwork was assessed by asking (I feel stressed about schoolwork; pressured, rushed not being able to keep up) answered in five options including always, often, sometimes (coded 1), seldom, and never (coded 0); **5**) bullying was assessed by asking for occurrences of bullying during the last three months and the question was explanatory with concrete examples of bullying (I have been bullied by other children in the last three months…), answered in two answer options including no (coded 1) and yes (coded 0); **6**) comfort in school (I am comfortable in school), was answered in five options including always, often (coded 1), sometimes, seldom, and never (coded 0); **7)** breakfast (I have breakfast) was assessed by a question about frequency of having breakfast during the school week with four answer options including daily (coded 1), 3-4 times/week, 1-2 times/week, and never (coded 0); **8**) physical activity/play (PAP) was assessed by asking how many occasions (1 occasion=60 min) per day/per week were spent on physical activity, exercising/playing until getting out of breath or sweaty (I have been physically active), answered in five answer options including daily 3-4 times/week (coded 1), 1-2 times/week, seldom and never (coded 0); **9**) smoking was assessed by asking for occurrence of smoking (smoking indoors at home), answered in four options including never and seldom (coded 1), sometimes and often (coded 0), and (I smoke) answered in five options

including never (coded 1), seldom sometimes, often, and always (coded 0); **10**) headaches, **11)** stomach aches and **12**) pains in back/neck/shoulders were assessed by asking for the frequency of severe headaches during the last three months, (I have had severe headaches, stomach ache, pains in back/neck/shoulders during the last three months), answered in five options including never and seldom, (coded 1), sometimes, often, and always (coded 0); **13**) feeling sad or depressed and **14)** worried or afraid during the three last months were assessed by asking (I have felt sad or depressed, worried or afraid) answered in five options including never and seldom, (coded 1), sometimes, often, and always (coded 0); **15**) feeling irritated or in a bad mood during the last three months were assessed by asking (I have felt irritated or in a bad mood) answered in five options including never and seldom, sometimes (coded 1), often, and always (coded 0); **16**) sleep (I sleep well), answered in five options including always, often (coded 1), sometimes, seldom, and never (coded 0).
